# Supplementary material for: Pyrocurrent anomalies and intrinsic magnetodielectric behavior near room temperature in Li2Ni2Mo3O12, a compound with distorted honeycomb and spin-chains
Source: Sci Rep. 2017 Jun 30;7:4449. doi: 10.1038/s41598-017-04025-9 (PMC5493632; doi:10.1038/s41598-017-04025-9)
Supplement: Supplementary file 1 — Pyrocurrent anomalies and intrinsic magnetodielectric behavior near room temperature in Li2Ni2Mo3O12, a compound with distorted honeycomb and spin-chains [file 41598_2017_4025_MOESM1_ESM.pdf]

Supplementary Information

Pyrocurrent anomalies and intrinsic magnetodielectric behavior near room temperature in $Li_2Ni_2Mo_3O_{12}$, a compound with distorted honeycomb and spin-chains

Sanjay Kumar Upadhyay, Kartik K Iyer, Smita Gohil, Shankar Ghosh, P.L. Paulose, and E.V. Sampathkumaran*

Tata Institute of Fundamental Research, Homi Bhabha Road, Colaba, Mumbai 400005, India

***Corresponding author: sampath@mailhost.tifr.res.in

X-ray diffraction data:

In this section, we show the x-ray diffraction pattern in Fig. S1 along with Rietveld refinement and fitted parameters. We used the same structural parameters as that employed in ref. 8.

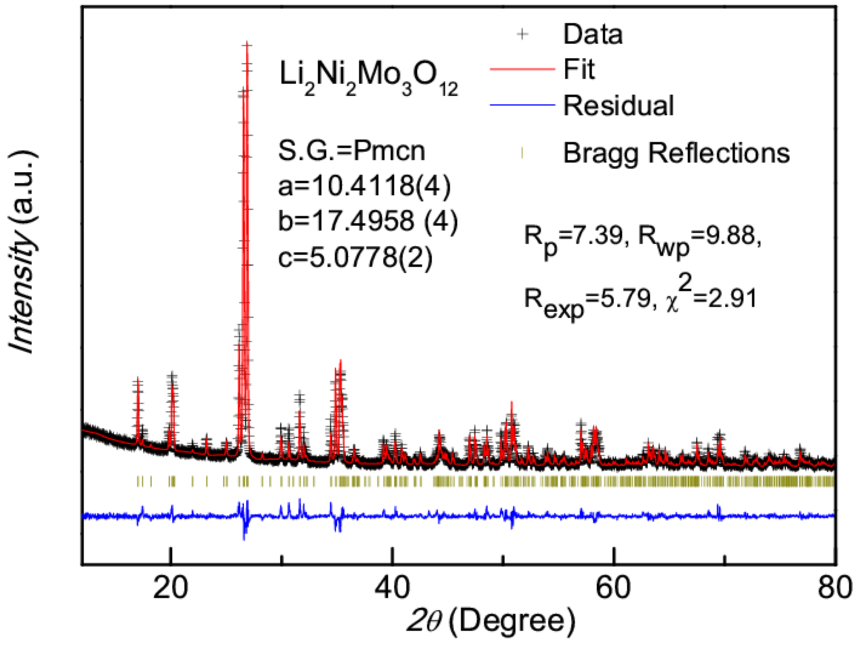

Supplementary Figure S1: XRD pattern (obtained at room temperature) along with the Reitveld refinement for $Li_2Ni_2Mo_3O_{12}$.

Magnetization data:

In order to show that the magnetization results obtained by us on this material are in good agreement with that reported in the literature, we show magnetic susceptibility curves and isothermal magnetization behaviour in Fig. S2. We made additional new findings, that is, the observation of a weak hysteresis around 40 kOe as well as the existence of a weak loop at low fields for 6 K.

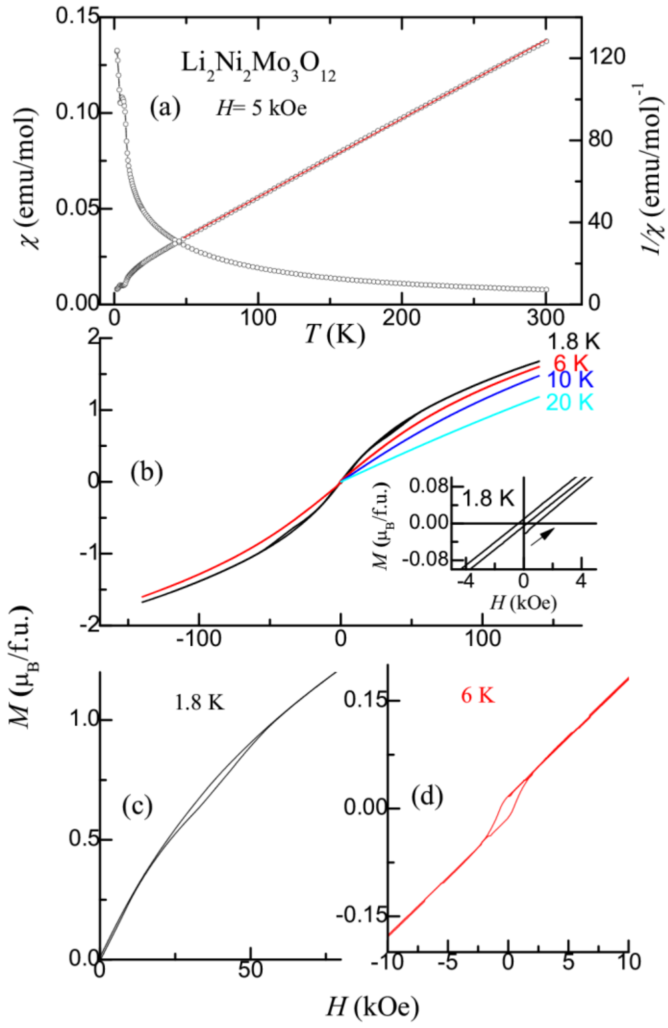

Supplementary Figure S2: (a) Magnetic susceptibility (χ) and inverse χ obtained in the presence of 5 kOe for $Li_2Ni_2Mo_3O_{12}$. In the inverse χ plot, the line drawn is obtained by Curie-Weiss fitting. In the case of χ versus *T* plot, a line is drawn through the data points to serve as a guide to the eyes. **(b)** Isothermal magnetization at selected temperatures. Top inset in (b) shows the *M(H)* at low fields at 1.8 K to highlight that the virgin curve lies outside envelope curve. (c) shows isothermal magnetization behaviour up to 70 kOe magnetic field at 1.8 K. (d) The M(H) curve in the low field region is shown in an expanded form for 6 K to highlight the existence of a low-field loop.

Pyrocurrent behavior:

In this section, we show the dependence of pyrocurrent peak on the rate of change of temperature. We have poled from 300 K to 2 K with the applied electric field of 4 kV/cm and collected the data for the different rates of warming (*dT/dt*=) 2 K/min, 3 K/min, 4 K/min and 5 K/min (see Fig. S3) from 2 K in independent measurements. We observe a peak around 300 K, but there is an observable rate dependence of the peak temperature.

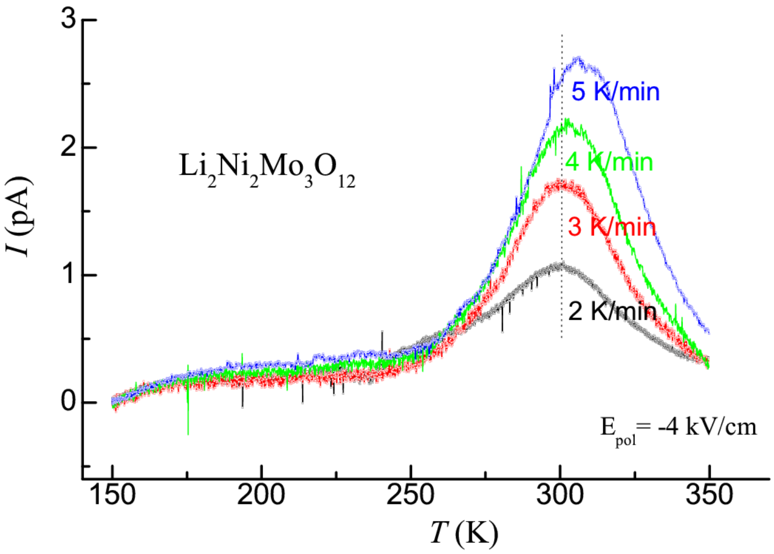

Supplmentary Figure S3: Pyrocurrent behaviour for $Li_2Ni_2Mo_3O_{12}$ for different heating rates. A dotted vertical line is drawn to show that the peak shifts with higher rate of heating.
